# Validating Single-Camera Pose Estimation Against Multi-Camera Motion Capture for Accessible Biomechanical Assessment

**DOI:** 10.1109/access.2026.3687207

**Published:** 2026-04-27

**Authors:** ANIKET PRATAPNENI, RYAN HALVORSON, PAVLOS SILVESTROS, NICHOLAS HARRIS, JEANNIE F. BAILEY

**Affiliations:** 1 Department of Orthopaedic Surgery, University of California, San Francisco, San Francisco, CA 94122, USA; 2 School of Medicine, University of California, San Francisco, San Francisco, CA 94131, USA; 3 Aperture LLC, Berkeley, CA 94702, USA

**Keywords:** Biomechanics, monocular pose estimation, markerless motion capture, joint kinematics, range of motion, error analysis, validation, gait and functional tasks

## Abstract

Motion analysis is critical for diagnosing and managing musculoskeletal disorders, but conventional multi-camera systems are expensive, limited to controlled environments, and require technical experience to operate. Single-camera, deep-learning-based pose estimation has emerged as a low-cost, portable alternative, yet its accuracy in clinical contexts remains underexplored. This study evaluated the accuracy of a single-camera pose estimation model (MeTRAbs) against a validated multi-camera system (THEIA3D) during gait, sit-to-stand, and trunk flexion-extension. 51 participants completed 669 movement trials recorded simultaneously with a smartphone camera (30Hz) and THEIA3D (180Hz). System outputs were aligned using similarity transforms. Root-mean-square errors (RMSEs) assessed positional accuracy for tracked joints and angular accuracy for computed sagittal joint angles (hips, knees, trunk). Intraclass correlation coefficients (ICCs) and Bland-Altman plots characterized reliability and systematic bias. Across all tasks, mean trajectory RMSE was 5.95 cm and joint angle RMSEs ranged 2.10°−10.98°. Stratifying errors per movement type and anatomical plane demonstrated that proximal joints and frontal plane motions showed higher fidelity, with the greatest errors in distal, dynamic joints (e.g., ankles in gait). Despite discrepancies, ROM ICCs exceeded 0.93 for all angles with a mean ICC of 0.967, indicating strong test-retest reliability. Errors were predominantly attributable to systematic bias, not random error, and harmonic correction substantially reduced angular error, confirming their predictable nature. These findings support single-camera pose estimation as a feasible, scalable tool for accessible motion analysis in rehabilitation and telehealth contexts. While not a full substitute for multi-camera capture, its accuracy and reliability suggest value for outpatient, telehealth, and rehabilitation contexts.

## I. INTRODUCTION

Quantitative motion analysis plays a central role in the assessment and treatment of musculoskeletal disorders. Traditionally, these analyses have relied on marker-based on multi-camera markerless motion capture systems, which offer high spatial and temporal fidelity. However, such systems require specialized and expensive equipment, dedicated and controlled capture environments, and substantial technical expertise, limiting access to high-fidelity motion capture in most orthopedic and rehabilitation settings. As a result, motion analysis is difficult to integrate into routine orthopedic care. Furthermore, as healthcare increasingly shifts toward decentralized and telemedicine-compatible modalities, there is a growing need for accurate, scalable, and cost-effective tools that can assess functional movement in real-world clinical contexts.

Recent advancements in computer vision and deep learning have given rise to a new class of motion capture tools that operate using only a single video camera or smartphone camera. These single-camera, markerless pose estimation models are trained to infer 3D joint positions from 2D video footage [1], [2], [3]. This approach would eliminate the need for multi-camera calibration and controlled studio environments, potentially enabling rapid, low-cost motion capture and full-body biomechanical analysis in the clinic or even at home, using smartphones or tablets that patients and clinicians have on hand. Furthermore, some of these models are notable for their ability to estimate metrically scaled 3D joint coordinates from single-camera input, removing the need for geometric calibration.

However, while promising, these approaches have primarily been assessed in broad performance settings such as athletics or general activity recognition and often lack joint- and task-specific comparisons against high-fidelity motion capture systems [4], [5], [6]. Other prior validation studies that did have a more rigorous approach often focused on isolated tasks or single joints [7], [8], [9]. Few have performed validation of single-camera pose estimation model performance on clinically relevant functional tasks, and those that have tend to report correlations between metrics derived from different joint tracking approaches rather than the degree of discrepancy between the tracking itself [10], [11]. Therefore, little is known about the reliability of single-camera pose estimation in clinically relevant tasks, such as sit-to-stand transitions, trunk flexion-extension, and gait, which represent functional movements that are routinely assessed in both operative and non-operative care to evaluate patients’ lower limb function, spinal mobility, fall risk, and so on.

Recent efforts have begun addressing this gap. Stenum et al. [9] validated video-based pose estimation for gait in clinical populations (Parkinson’s disease and post-stroke), though their analysis emphasized correlation between spatiotemporal parameters rather than direct kinematic discrepancies. Horsak et al. [12] reported monocular mean joint angle errors of 5.4° during physiological gait in 19 healthy participants but focused solely on joint angles and did not further characterize error structure. Rode et al. [13] analyzed a variety of physical activities and demonstrated starkly increased error in 3D reconstruction relative to 2D (14.6–24.9 cm versus 7.2–12.2 cm) but focused only on mean per joint position error and isolated knee and elbow flexion angle errors without task stratification. While these studies demonstrate feasibility, systematic characterization of error structure across tasks, joints, and movement planes – essential for understanding when and how single-camera systems can substitute for laboratory-based capture – remains limited.

This leaves open questions about whether single-camera inference can reliably capture dynamic, multi-planar motion in complex tasks, particularly when accuracy is needed for downstream biomechanical modeling or patient specific diagnostics. To meet this need, we propose a comprehensive assessment of single-camera pose estimation performance, benchmarked against a validated, high-fidelity, multi-camera motion capture system. Our study addresses current gaps by: (1) evaluating multiple clinically relevant tasks within a single cohort to enable direct performance comparison, (2) decomposing trajectory error by anatomical plane to identify systematic sources related to monocular depth ambiguity, and (3) demonstrating the correctable versus random components of error through harmonic regression analysis.

The purpose of this study is to evaluate the accuracy of single-camera pose estimation across multiple joints and tasks of orthopedic relevance, and to identify specific contexts in which this approach excels or falls short. Single-camera systems’ accuracy can be limited by both random and systematic sources of error. Random error is known to arise from transient occlusion of joints during motion and dynamic extremity movement. Systematic error may result from the depth ambiguity inherent to single-camera capture: while frontal plane motion is accurately captured, depth must be inferred. Other potential sources of systematic error are fixed biases in the model architecture, such as differences in predicted joint marker geometry and from training datasets that rarely contain orthopedic mobility assessments [4], [12], [13], [14], [15], [16], [17], [18]. Based on these limitations, we hypothesized that pose estimation would demonstrate comparable accuracy to multi-camera motion capture in proximal joints and movements predominantly in the camera’s frontal plane, but would suffer larger deviations in distal, dynamic joints, particularly in tasks involving greater motion or self-occlusion. Understanding the nature of these deviations and the relative contributions of random and systematic error is essential for interpreting single-camera performance and guiding post hoc correction strategies.

## II. MATERIALS AND METHODS

### A. SUBJECTS

In total, 51 subjects (24 male, 27 female; age 51.5 ± 16.7 y) participated in this study, including 31 healthy individuals and 20 chronic low back pain patients. All subjects provided written informed consent prior to participation, and the study was approved by the UCSF Institutional Review Board (Protocol #20–31485).

### B. MEASUREMENT

#### 1) MODEL SELECTION

MeTRAbs – a deep learning-based single-camera 3D pose estimation model [3] – was selected for its capacity for metric-scale joint localization and robustness to camera positioning and occlusion. Among existing pose estimation models, MeTRAbs was chosen for its strong generalization performance on diverse datasets and its ability to output joint positions in absolute 3D space without requiring additional calibration or cameras, making it well-suited for comparison with motion capture data.

While newer pose estimation models have emerged since MeTRAbs’ release, we selected it for this validation because: (1) it provides metric-scale 3D coordinates without calibration, essential for direct comparison with motion capture; (2) it has documented robustness to varying camera positions; and (3) it represents a widely-accessible, open-source baseline that practitioners can readily deploy. This validation establishes performance benchmarks applicable to similar metric-space pose estimation approaches.

High-fidelity motion data was collected using an 8-camera THEIA 3D system, which combines high spatial resolution with the practical advantage of markerless capture. THEIA has been validated against marker-based systems [21], [22], allowing for less obtrusive data collection while maintaining a high degree of kinematic fidelity.

In all three movements, the camera was placed approximately 3 meters away from the participant to capture the full body in frame, at an offset from midline (anterior-oblique in sit-to-stand and trunk flexion-extension and posterior-oblique in treadmill gait). We did not use an exact measurement tool when setting up the camera but always kept it in a specific area relative to the participant that is estimated to be roughly 10–20° to the right of the sagittal plane. We allowed for some variation in the specific camera distance and angle in order to make our analysis more robust and generalizable to practical use.

#### 2) GAIT TRIALS

Participants walked on a treadmill at a self-selected speed for up to 11 minutes. Trials were terminated early if participants self-reported a pain intensity of 7/10 or greater, or if pain increased by 4 points relative to baseline (numeric rating scale collected every minute).

Continuous video of each trial was recorded using a single camera (iPhone, Apple, California, USA) sampling at 30 Hz, positioned to capture full-body movement. These videos served as input for the MeTRAbs pose estimation model to obtain 3D joint trajectories. At the onset of steady-state walking (designated as time zero), a 15-second segment was also captured using an 8-camera markerless motion capture system (THEIA3D, sampling rate: 180 Hz), and this was repeated every minute.

#### 3) TRUNK FLEXION-EXTENSION TRIALS

Participants were instructed to perform three full cycles of forward flexion and backward extension while standing. The entire movement sequence was captured simultaneously by both the single-camera video and multi-camera markerless motion capture.

#### 4) SIT-TO-STAND TRIALS

Participants performed five consecutive sit-to-stand movements, beginning from a seated position. The entire movement sequence was captured simultaneously by both the single-camera video and the multi-camera markerless motion capture.

### C. DATA PROCESSING

To minimize downstream effects of measurement noise as much as possible, signal filtering was applied before any alignment or trajectory comparison. All pose data – from both the single- and multi-camera systems – were first subjected to a Savitzky-Golay filter (window length: 0.5 s, polynomial order: 3) to smooth joint position trajectories and reduce frame-to-frame jitter without losing high-frequency components of motion [23]. This window length was selected to preserve gait cycle dynamics while attenuating high-frequency noise from the 30 Hz smartphone camera. Sensitivity analysis with window lengths of 0.3s and 0.7s showed minimal impact on RMSE values (<0.5 cm across all joints), confirming robustness of our results to this parameter choice.

Unlike simple moving average or low-pass filters, which attenuate peak amplitudes and can shift peak timing due to distortion, Savitzky-Golay performs a local polynomial regression within a sliding window, which preserves the local shape of the signal, including peak magnitude and curvature, while reducing high-frequency noise. Preservation of these features was particularly important because angular velocity and peak kinematic events are clinically relevant and filters that excessively smooth the signal can dampen such derivative features. The Savitzky-Golay filter was therefore selected specifically for its ability to maintain trajectory geometry, permit more accurate estimation of derivatives, and eliminate phase distortion. Wavelet denoising was also considered for its excellent peak preservation and multi-scale denoising, but this filter demands more complex parameter selection and is more computationally intensive without offering the same derivative accuracy.

To align trajectories, we applied a similarity transformation (isotropic scaling, rotation, and translation) computed separately for each trial. This Procrustes-style alignment minimized the sum of squared distances between corresponding joint positions in the mean pose (averaged across all frames of that trial). Alignment was performed on a per-trial basis to account for differences in subject positioning and distance from the single-camera versus the multi-camera setup. While this alignment approach reduces absolute positional error, it is necessary for fair comparison. This means our reported RMSEs represent relative motion accuracy rather than absolute global positioning error and parallax error caused by differences in camera orientation, which may be higher in uncalibrated real-world deployment. Importantly, alignment does not affect relative motion patterns or joint angle calculations, which were the primary outcome metrics of interest.

Key sagittal joint angles (hip, knee, trunk) were computed from 3D joint positions using a vector-based approach. For each joint, local segment axes were constructed to define the angle in the sagittal plane, allowing comparison of kinematic trends between the two datasets.

### D. DATA ANALYSIS

To evaluate the similarity between single-camera inference joint trajectories and those obtained from the multi-camera markerless motion capture system, the root-mean-square error (RMSE) was calculated on a frame-by-frame basis for each corresponding joint trajectory in 3D space, after spatial alignment and downsampling the motion capture data to match framerates. This provided a straightforward measure of pointwise deviation between the two modalities.

For additional validation, we employed dynamic time warping (DTW) to identify potential small-scale temporal misalignments between the single- and multi-camera recordings that may have occurred at the time of data collection [24]. DTW facilitates comparison of trajectories that are similar in shape but may be out of phase by finding the optimal non-linear alignment path that minimizes the total Euclidean distance between corresponding points. This algorithm was therefore used to warp the single-camera inference trajectories to match the temporal structure of the multi-camera motion capture data and then calculate the mean temporal deviation per frame (in number of frames off from the motion capture data). Trials in which the DTW alignment path deviated significantly from the diagonal (mean absolute deviation of over 15 frames, or 0.5 seconds), indicating excessive temporal warping due to temporal misalignment at the time of data collection, were flagged and excluded from statistical analysis, as they likely reflected desynchronization of recording initiation at the time of data collection. 16 trials were excluded, out of a total of 669.

### E. STATISTICAL ANALYSIS

For each joint, the 3D RMSE was calculated, along with 95% confidence intervals, obtained via nonparametric bootstrap resampling (10,000 iterations). To evaluate differences in pose estimation performance across the three movement types evaluated, we used mixed-effects models accounting for repeated measures within participants, with movement type as a fixed effect and participant as random effect. Post-hoc pairwise comparisons between movement types used t-tests for pairwise comparisons. Bonferroni correction (adjusted *α* = 0.05/3 = 0.017) was used for three pairwise comparisons. Intraclass correlation ICC(3,1) coefficients were calculated to assess inter-method consistency for joint ranges of motion. Note that ICC reflects agreement rather than test-retest reliability in this context. Pearson r was used to test for the relationship between BMI and joint tracking error magnitude.

### F. POST-HOC HARMONIC CORRECTION

To assess the systematic nature of angle errors, we fitted a dual-frequency harmonic regression model to the error time series for each joint angle:

$$\text{error}\;\left(t\right)=A_{1}\cdot \text{sin}\left(\omega t+\varphi_{1}\right)+A_{2}\cdot \text{sin}\left(2\omega t+\varphi_{2}\right)+\varepsilon$$

where *ω* represents the fundamental frequency of the movement cycle. Correction parameters were derived from the mean error function across all trials of each movement type (not individualized per participant). To assess whether these correction parameters were generalizable, we performed 5-fold cross-validation. Trials were partitioned into five folds: this was done on a per-participant basis rather than a per-trial basis to prevent trials from the same participant being present in both the training and validating data. For each fold, harmonic parameters were derived from the remaining four folds and applied to the held-out fold. We report both within-sample (all trials) and cross-validated correction performance to demonstrate proof-of-concept for systematic bias removal.

## III. RESULTS

In this study, 51 participants – 24 males and 27 females – with an average age of 51.5 ± 16.7 y performed a total of 432 treadmill gait trials, 96 sit-to-stand trials, and 141 trunk flexion-extension trials. Mean BMI was 25.8±4.6 kg/m^2^ (range: 18.0–40.1). Among the 23 low back pain patients, mean pain intensity at baseline was 3.70±2.03 on a 0–10 numeric rating scale (range: 1–8).

### A. GENERALIZED POSE RESULTS

Quantitative analysis demonstrated that the pose inference model applied to single-camera recordings exhibited strong agreement with the trajectories tracked by the multi-camera motion capture system. With each movement type weighted equally, mean RMSE across all joint trajectories was 5.95 cm, with the highest mean RMSE observed at the right ankle (7.65 cm), suggesting consistently high level of fidelity in positional inference across the body. On a per-participant basis, the variance in average RMSE across joints was 0.04 cm, indicating that model performance was robust to the intersubject variability in body habitus, limb length, and morphology.

### B. JOINT-SPECIFIC VALIDATION

There was no significant difference between individuals with and without low back pain across all joints (all p > 0.05). There was no relationship between participant BMI and RMSE for any joint (all p > 0.05, all Pearson r < 0.06), indicating that the single-camera approach was not less robust to BMI or pain-limited movement than the multi-camera approach.

Error magnitude varied across joints, with proximal landmarks generally exhibiting lower RMSEs than distal ones (Table 1). Most significantly, the ankles displayed a much higher error (7.47 cm and 7.65 cm) than all other keypoints (p < 0.01). Among the joint angles analyzed (Table 1), the highest RMSE was observed in right knee flexion (10.98°), while hip and shoulder axial rotation angles (internal and external rotation) exhibited significantly lower errors (2.10° and 2.60°, respectively) than other angles (p < 0.01 for both), suggesting that the model can estimate rotational kinematics when the rotation axis is approximately aligned with the camera viewing direction. Dimensional decomposition of trajectory error revealed that the model performed worst in the axial plane, followed by the sagittal plane, which accounted for 44.02% and 34.98% respectively of the total MSE across joints. This dimensional error contribution was relatively constant across all joints (Table 2).

### C. TASK-SPECIFIC VALIDATION

Stratifying results by movement type failed to identify any statistical difference in error magnitude between individuals with and without low back pain (p > 0.05 for all joints in all movements). Similarly, stratification did not reveal relationship between BMI and RMSE for any joint (all p > 0.05, greatest Pearson r = 0.26 for the right ankle in trunk flexion-extension), suggesting robustness to modest clinical heterogeneity and body habitus.

**FIGURE 1.**
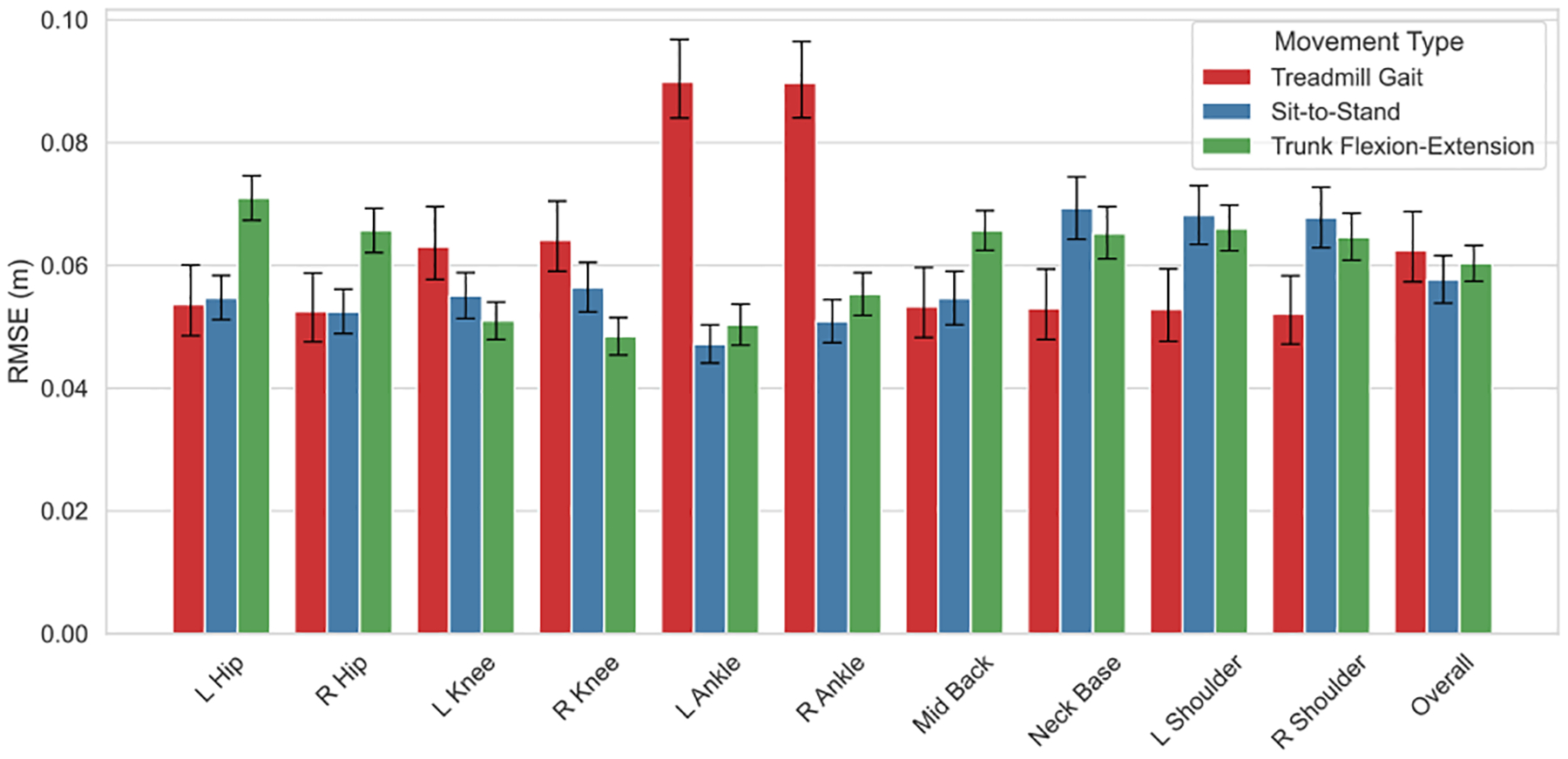
RMSE between 3-dimensional trajectories of joints in single- and multi-camera output, averaged across all participants and trials for each movement task (gait, sit-to-stand, and trunk flexion-extension). Error bars represent 95% confidence intervals.

The single-camera inference model demonstrated robust performance across all movement types, including treadmill walking, trunk flexion-extension, and sit-to-stand transitions. As expected, joints undergoing greater displacement showed higher errors (Fig. 1, Table 3). During treadmill walking, for instance, ankle RMSEs (8.83 cm and 8.86 cm) were higher (p < 0.01) than those of the knees (6.26 cm and 6.43 cm), which in turn exceeded those of the hips (5.34 cm and 5.23 cm, p < 0.01). A similar pattern was observed in sit-to-stand transitions, where errors were significantly (p < 0.01) larger at the neck (6.84 cm) and shoulders (6.73 cm and 6.70 cm) than at the more static keypoints like the ankles (4.68 cm and 5.06 cm) and knees (5.46 cm and 5.60 cm). Trunk flexion-extension tasks exhibited a similar pattern of error to sit-to-stand, but with increased errors at the hips as well (7.04 cm and 6.51 cm, p < 0.01).

Interestingly, when normalized to the joint’s range of motion (ROM), this pattern was inverted (Table 4). Dynamic joints such as the ankles during treadmill walking (12.22% and 12.23%), the shoulders during sit-to-stand (9.79% and 9.73%), and the neck base during trunk flexion-extension (11.44%) exhibited lower ROM-normalized RMSEs, reflecting accurate relative tracking despite larger absolute errors. In contrast, static or minimally moving joints showed larger ROM-normalized RMSEs. The most striking examples of this were the ankles during planted tasks (sit-to-stand and trunk flexion-extension), which showed ROM-normalized RMSEs exceeding 100%, driven by small absolute errors occurring over negligible true motion ranges.

The trend observed in absolute RMSEs was further corroborated by the errors in joint angles: treadmill walking was characterized by higher errors in knee flexion angles, sit-to-stand transitions by higher errors in knee flexion and hip flexion angles, and trunk flexion-extension by higher errors in hip flexion and trunk inclination angles (Table 5). Of note, trunk inclination errors displayed a clear stepwise increase in magnitude from treadmill (2.85°) to sit-to-stand (8.75°) to trunk flexion-extension (14.41°).

**FIGURE 2.**
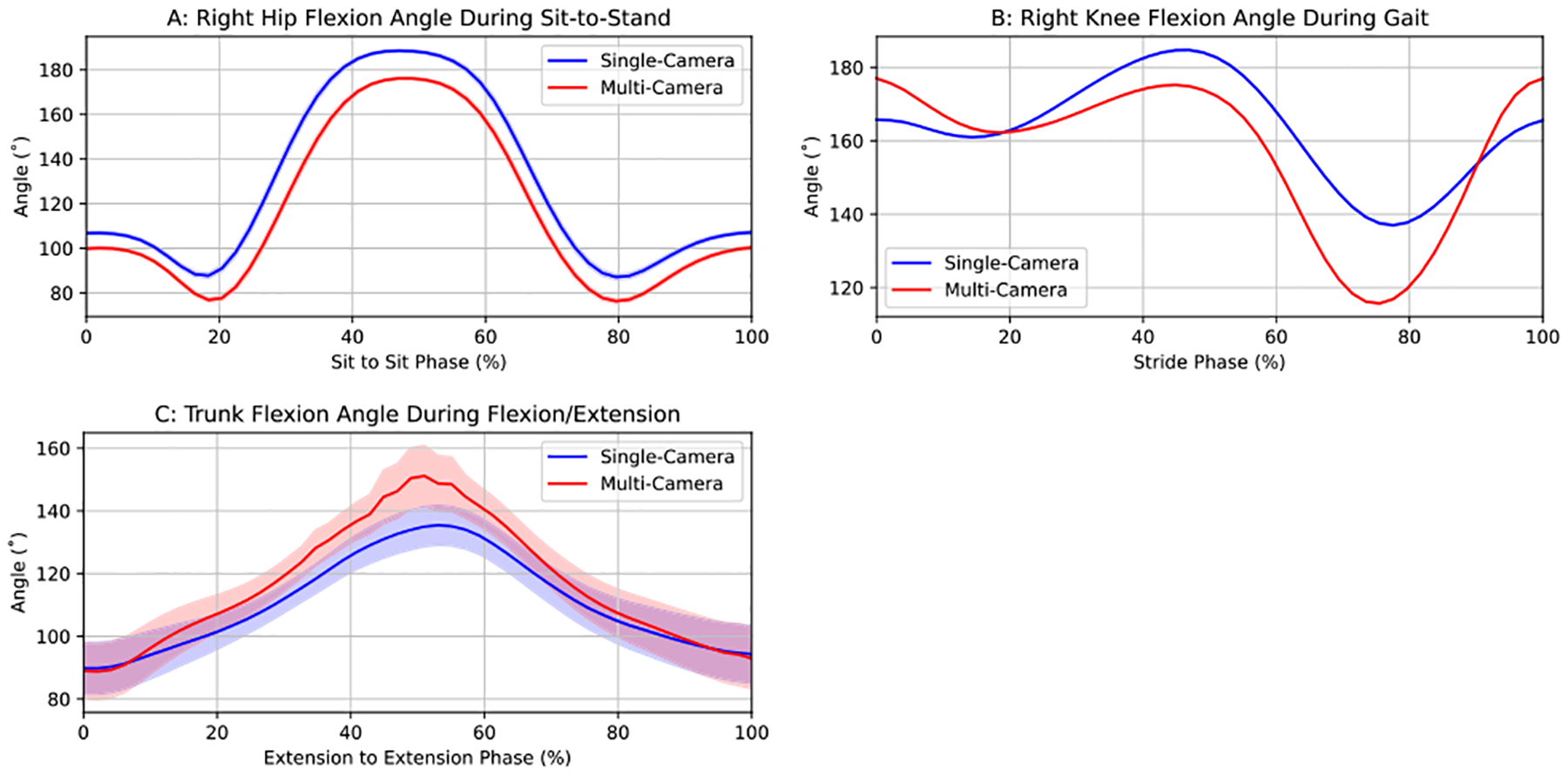
Comparison of mean joint angle trajectories from single- and multi-camera output for the most error-prone angles of each movement type across normalized movement cycles, with 95% confidence bands (these are narrow and difficult to visualize in panels A and B due to low inter-trial variability).

However, despite the magnitude of these errors, overlay plots of joint angles that showed the greatest error for each movement (Fig. 2) revealed that both systems captured very similar trajectory shapes across trials. Mean trajectories followed the same temporal pattern, and the corresponding 95% confidence bands were narrow for both systems, indicating low intra-condition variability. This suggests that the observed discrepancies may stem from consistent, joint-specific biases due to camera position and the underlying keypoint geometry rather than random error.

**FIGURE 3.**
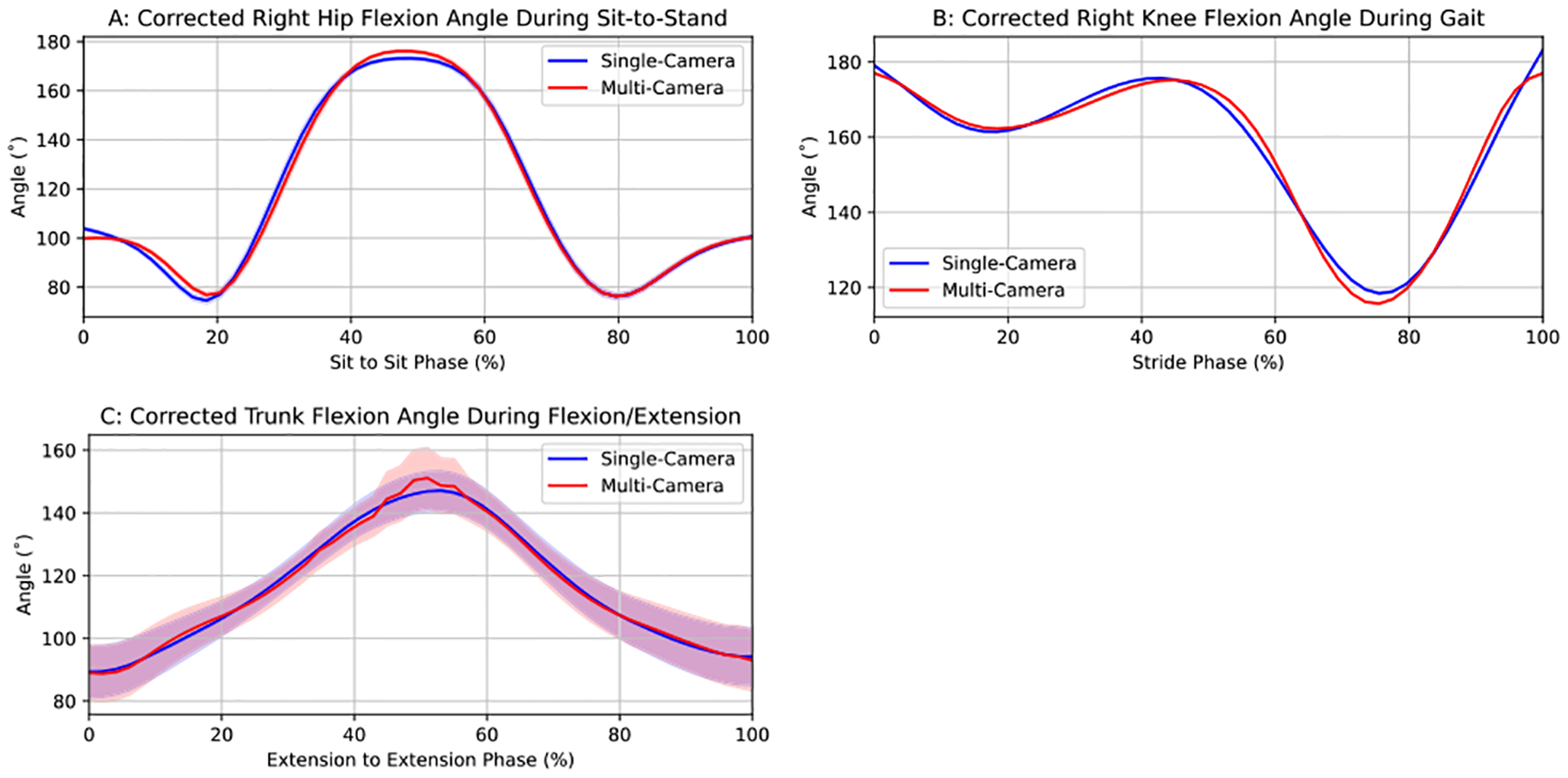
Comparison of mean joint angle trajectories from single- and multi-camera output with post hoc harmonic correction for the most error-prone angles of each movement type across normalized movement cycles, with 95% confidence bands (these are narrow and difficult to visualize in panels A and B due to low inter-trial variability).

This is further corroborated by overlay plots of the same high-error joint angles with a correction factor that was determined by performing a dual-frequency harmonic regression on the error functions (Fig. 3). Qualitatively, there is a near-perfect agreement in the joint angle trajectories, and quantitatively, the mean absolute error decreased significantly: 12.12° to 4.42° in right hip flexion during sit-to-stand, 11.24° to 5.28° in right knee flexion during gait, and 9.72° to 8.05° in trunk inclination during trunk flexion-extension. Cross-validated correction (5-fold) yielded near-identical reductions: 12.11±1.19° to 4.48±0.73° in right hip flexion during sit-to-stand, 11.26±0.56° to 5.34±0.24° in right knee flexion during gait, and 9.73±1.50° to 8.24±1.44° in trunk inclination during trunk flexion-extension, confirming that correction parameters generalize to unseen trials under the same experimental conditions. This indicates that the systematic bias arising from camera position and marker geometry may be addressable via post hoc correction or calibration.

### D. JOINT RANGE OF MOTION

**FIGURE 4.**
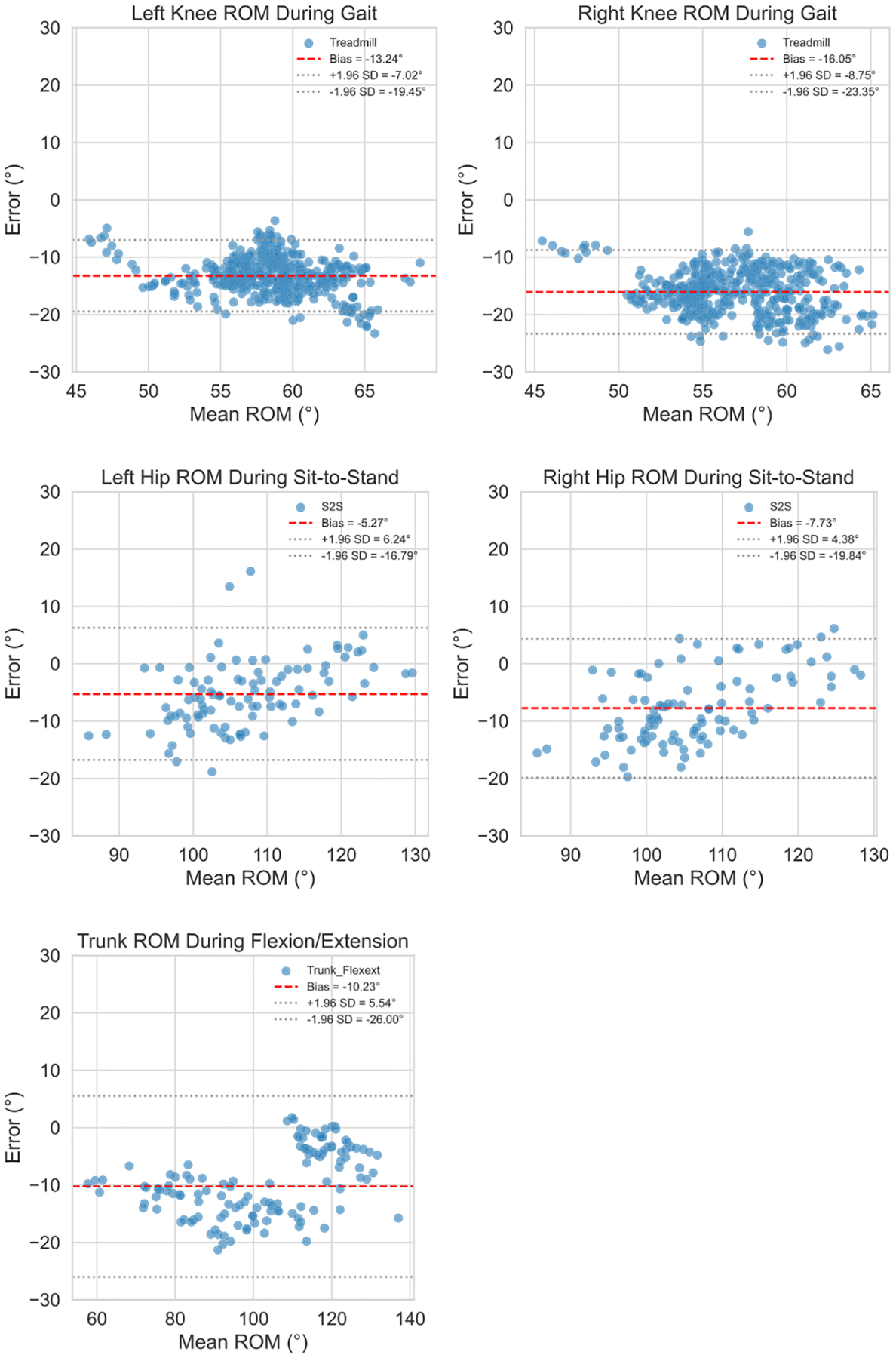
Bland-Altman analyses of ROM agreement between single- and multi-camera systems for the most error-prone joint angles in each movement type.

To further evaluate the clinical relevance of single-camera pose estimation, joint ranges of motion (ROM) derived from the two systems were compared. In agreement with previous error patterns, the largest mean absolute error was observed in the most dynamic joints: knees in gait (14.65°), hips in sit-to-stand (7.51°), and trunk inclination in trunk flexion-extension (10.31°). Bland-Altman analyses (Fig. 4) revealed systematic differences between methods that closely mirrored the mean absolute errors (e.g., −14.65° for knee flexion in treadmill gait, −6.50° for hip flexion in sit-to-stand, and −10.23° for trunk inclination in trunk flexion-extension), suggesting the discrepancy is largely attributable to a consistent underestimation that might be corrected for, rather than random noise. Additionally, the intraclass correlation coefficients ICC(3,1) for ROM estimates were very high (>0.9) across all joint angles and tasks, indicating strong consistency in the relative magnitudes of motion captured (Table 6). This suggests that, in terms of ranking or broadly quantifying mobility, the single-camera system may offer clinically acceptable precision.

While the spans of the 95% limits of agreement (LoA) for the key angles mentioned above indicated that absolute agreement with multi-camera motion capture did vary meaningfully across trials, that variation did not follow any discernible trend – the error occurred uniformly across all observed ROMs, supporting the model’s use for longitudinal tracking and relative comparisons rather than absolute quantification in isolation.

## IV. DISCUSSION

This study evaluated the accuracy of MeTRAbs, a single-camera deep learning-based pose estimation algorithm, by comparing its outputs from inference on smartphone videos to those of a validated multi-camera markerless motion capture system (THEIA 3D) across multiple clinically relevant movement tasks. Although there is no universally accepted threshold for clinically significant error in pose estimation, our analysis emphasizes comparability across systems rather than absolute perfection. In this context, we sought to identify whether single-camera pose estimation can serve as a practical, reliable, and scalable tool for orthopedic motion analysis, and to pinpoint specific scenarios in which its use is most appropriate.

Few prior studies have used RMSE to evaluate 3D joint tracking accuracy of single-camera pose estimation algorithms in clinical contexts. However, across a set of 16 state-of-the-art human pose estimation methods, the mean per joint positional error (MPJPE) in joint tracking ranged between 4.65 cm and 7.19 cm, with an average MPJPE across studies of 5.60 cm [3], [23], [24], [25], [26], [27], [28], [29], [30], [31], [32], [33], [34], [35], [36], [37]. Our MPJPE of 5.95 cm falls within this range, suggesting that this approach can achieve low-centimeter root-mean-square error (RMSE) in 3D joint localization and under 11° error in joint angle estimation for major anatomical landmarks and kinematically important metrics.

More relevant for clinical interpretation are validation studies using similar functional tasks. Prior study has shown strong correlations (r > 0.9) between video-based and marker-based gait spatiotemporal parameters but did not quantify and stratify joint angle or trajectory errors directly [9]. Another study reported monocular mean per joint angle errors of 5.4° [12], significantly lower than our findings, though their camera-extrinsic calibration may account for this difference. A third study found that mean per joint error was significantly higher in 3D reconstruction (14.6–24.9 cm) than 2D estimation (7.2–12.2 cm) [13], corroborating our plane-wise error decomposition findings. Notably, few prior clinical validations have systematically characterized error by anatomical plane or quantified systematic versus random error components as we have done here. Our finding that 44% of positional error occurs in the axial plane directly confirms the depth ambiguity limitation of monocular capture, while our harmonic correction analysis demonstrates that these systematic biases are predictable and potentially addressable through post-hoc calibration. Our findings thereby support single-camera pose estimation’s potential utility for biomechanical assessment and monitoring in remote or resource-limited clinical settings, particularly when the goal is to broadly quantify or capture changes in mobility.

Overall, MeTRAbs showed the highest fidelity at proximal joints and static/minimally mobile regions, with error increasing at more distal or highly dynamic joints. Notably, the ankle RMSEs exceeded 7.5 cm on average across all tasks and reached nearly 9.0 cm during treadmill gait. This is consistent with prior findings that suggest pose estimation models struggle with rapid, small-radius movements at the extremities due to motion blur, occlusion, and error amplification along kinetic chains [15], [20]. Another potential explanation for the heightened ankle error may be model geometry: if the ankle marker of the pose estimation model was defined more proximally or distally than the ankle marker of the motion capture system, the trajectories defined by the two would trace different arcs – particularly in gait – resulting in consistent RMSE inflation. In contrast to the model’s performance on ankles, hip positions and trunk inclination angles exhibited relatively low RMSEs, indicating better model performance in estimating large, centralized whole-body movement patterns and postural changes.

A similar trend emerged when comparing across movement types. Treadmill walking produced the highest joint trajectory RMSEs, especially at the ankles and knees, likely due to the increased translation and impact forces involved. Sit-to-stand transitions showed moderate error, while trunk flexion-extension, despite being slower and more spatially constrained, introduced error at the pelvis and spine, likely due to the fact that these movements are predominantly along the anteroposterior axis, which is difficult to capture with a single-camera view, as noted in prior validations [14], [16], [18]. The fundamental depth ambiguity inherent to monocular vision is further reflected by the disproportionately small contribution of coronal plane error (21.00%) to total MSE. Inferring position along the axis of the camera’s viewing direction requires the model to resolve depth from monocular cues such as relative size and learned body proportions, making the plane orthogonal to that axis relatively less error-prone. These task-specific differences, in conjunction with the distribution of plane-wise contributions to error, reflect not only biomechanical complexity but also the training limitations of pose estimation networks.

Interestingly, when the RMSE was normalized to each joint’s ROM, the pattern of performance was inverted. Joints with large motion arcs showed low relative errors (~10%), while joints that were relatively static in a given task (e.g., the ankles during trunk flexion-extension) exhibited normalized RMSEs exceeding 150%. This highlights an important interpretative caveat: absolute RMSE may overstate model underperformance in highly dynamic joints, while normalized RMSE can appear inflated in joints with near-zero true joint displacement. It is therefore important to consider these metrics together, to provide complementary perspectives on clinical usability.

The results of this validation suggest that single-camera pose estimation models, specifically metric-space models like MeTRAbs, are approaching clinically actionable accuracy levels in certain use cases. While multi-camera systems like THEIA remain the gold standard in high-fidelity motion analysis, the ability of MeTRAbs to deliver joint trajectory accuracy within 6–7 cm and joint angle accuracy under 11° makes it a compelling option for environments where full-rig motion capture is impractical or cost-prohibitive, such as longitudinal rehabilitation monitoring, gait screening in primary care, or functional movement assessments in rural or telehealth settings.

The findings for joint angles and ROMs – clinically significant metrics – were nuanced. Bland-Altman analysis suggested that the mean absolute error in ROM estimation was largely attributable to systematic differences between the two approaches, such as differences in marker geometry or camera position, but also revealed a degree of variation in the absolute agreement with multi-camera motion capture. Our analysis also demonstrated that the discrepancy between time-series joint angles estimated from single- and multi-camera approaches was reproducible and was well-corrected for by sinusoidal functions of stride phase, further supporting the conclusion that errors were largely attributable to systematic differences as opposed to measurement inaccuracies. Five-fold cross-validation demonstrated that correction parameters derived from training folds maintained performance on held-out trials, indicating that systematic biases are reproducible across the dataset. We would expect correction parameters to be sensitive to camera position, so prospective deployment of this technique would still require either individual calibration trials or population-level correction parameters. Nevertheless, these results suggest that such correction approaches are feasible and not simply overfitting to noise.

To contextualize these errors clinically, we note that minimal clinically important differences (MCID) for ROMs in osteoarthritis populations are estimated at 3.8–6.4° for knee flexion and 5.0° for hip flexion [40], [41]. While this population is dissimilar from the study population, it may provide useful context given the lack of established MCID in chronic low back pain patients. Our observed RMSEs of 5.29–12.48° for knee flexion and 6.93–15.40° for hip flexion thus approach or exceed these thresholds, indicating that absolute single-timepoint measurements may not yet achieve the precision required for definitive clinical decision-making – caution is warranted when interpreting absolute ROM values in isolation or when comparing metrics between different models that might have different marker geometries. However, for longitudinal monitoring where within-subject change is the focus, the high ICC values (>0.93) and systematic nature of errors suggest the system can reliably detect changes exceeding ~10–15° in ROM.

While our validation focused specifically on MeTRAbs, we expect these findings to generalize to other metric-scale pose estimation that output absolute 3D coordinates without calibration. Newer models may achieve lower absolute error, but we expect the fundamental challenge of depth ambiguity to persist. Thus, our characterization of systemic biases by anatomical plane, joint location, and movement type provides a useful benchmark for evaluating any single-camera system. Future comparative studies across multiple pose estimation architectures would help establish whether observed error patterns are model-specific or intrinsic to the monocular approach.

Some limitations warrant discussion. The 30 Hz smartphone framerate, while typical for consumer devices, may inadequately capture extremely rapid movements such as impact transients or high-velocity limb motions – these drawbacks are likely captured in our findings, comparing against a multi-camera modality with a 180 Hz framerate.

Additionally, treadmill gait differs from overground walking in subtle kinematic features [42], potentially affecting generalizability.

Our participant population was demographically heterogeneous and included both healthy controls and patients with low back pain, but clinical pathology and etiology were not specifically stratified, although based on the nature of the algorithm’s frame-by-frame estimation and the results of the comparison between low back pain patients and healthy controls presented here, we expect pose estimation to be robust to pathology.

We assessed within-session reliability but the single-timepoint nature of our study precluded evaluation of inter-session repeatability, which would be important for longitudinal monitoring applications.

Kinetic variables like joint velocity, ground reaction force, and acceleration are often more clinically informative but were not analyzed. These variables may be more sensitive to positional error than raw kinematics, as differentiation amplifies signal noise. Due to the dependence of kinematic variables on positional data, we expect their error characterization to be similar, but future study is warranted to test this hypothesis. Practitioners should therefore exercise caution when using single-camera pose estimation for applications requiring accurate kinetic estimates or inverse dynamics modeling.

Our single camera viewpoint (posterior-oblique in gait and anterior-oblique otherwise, 10–20° offset) had limited variability, and precise camera angles were not recorded, precluding formal sensitivity analysis of positioning effects on accuracy. Performance may vary with lateral or anterior camera positions, which should be explored in future work.

A critical methodological consideration is that this represents markerless-to-markerless validation. While THEIA has been validated against marker-based systems [21], [22], it is itself a markerless method. This introduces the possibility of shared failure modes between THEIA and MeTRAbs in scenarios involving severe occlusion, ambiguous poses, or particularly complex motion. This could result in underestimation of true error relative to a marker-based system. However, it is important to note that marker-based systems also require a kinematic estimation model to compute joint center positions from joint surface markers, which means it may be impossible to entirely eliminate the possibility of shared failure modes between skeletal tracking approaches. Regardless, future work should include direct comparison against marker-based systems to fully characterize accuracy and provide generalizability to a wider range of current gold standard skeletal tracking approaches.

## V. CONCLUSION

This study provides a comprehensive evaluation of MeTRAbs single-camera pose estimation across multiple functional tasks and anatomical landmarks. While not without limitations, the method achieves promising accuracy in 3D joint localization and subsequent joint angle calculation, particularly for centrally located joints and in postural tasks. The results support its potential as a clinically viable, scalable alternative to traditional motion capture for certain clinical applications, especially in settings that demand accessibility, portability, or scalable rapid deployment.

## Figures and Tables

**TABLE 1.** Positional and Angular RMSE between 3-dimensional joint trajectories and key joint angles from single- and multi-camera output, averaged across all trials and participants.

Keypoint	RMSE [95% CI] (cm)
Left Hip	5.67 [5.26, 6.17]
Right Hip	5.46 [5.06, 5.95]
Left Knee	5.91 [5.50, 6.41]
Right Rnee	5.99 [5.59, 6.49]
Left Ankle	7.47 [7.01, 8.02]
Right Ankle	7.65 [7.20, 8.18]
Mid-Back	5.55 [5.15, 6.04]
Neck Base	5.71 [5.30, 6.21]
Left Shoulder	5.71 [5.28, 6.22]
Right Shoulder	5.63 [5.23,6.11]
Overall	6.07 [5.67, 6.57]
Joint Angle	RMSE [95% CI] (degrees)
Left Knee Flexion	8.45 [8.01, 8.94]
Right Knee Flexion	10.98 [10.52, 11.47]
Left Hip Flexion	8.98 [8.55, 9.46]
Right Hip Flexion	10.61 [10.22, 11.04]
Hip Internal/External Rotation	2.10 [1.89, 2.37]
Shoulder Internal/External Rotation	2.60 [2.39, 2.88]
Trunk Inclination	5.98 [5.58, 6.38]

**TABLE 2.** MSE in each anatomical plane expressed as a percentage of total MSE, averaged across all trials and participants.

Keypoint	Sagittal Plane	Coronal Plane	Axial Plane
Left Hip	33.99	23.22	42.78
Right Hip	33.31	23.39	43.29
Left Knee	36.51	16.91	46.58
Right Knee	37.30	16.60	46.09
Left Ankle	40.20	16.62	43.18
Right Ankle	40.56	17.41	42.03
Mid-Back	32.33	25.40	42.27
Neck Base	31.73	22.69	45.58
Left Shoulder	31.45	24.48	44.07
Right Shoulder	32.45	23.22	44.33
Overall	34.98	21.00	44.02

**TABLE 3.** RMSE between 3-dimensional trajectories of joints in single- and multi-camera output.

Keypoint	Treadmill Gait Mean RMSE [95% CI] (cm)	Sit-to-Stand Mean RMSE [95% CI] (cm)	Trunk Flexion-Extension Mean RMSE [95% CI] (cm)
Left Hip	5.34 [4.74, 6.09]	5.40 [5.06, 5.73]	7.04 [6.69, 7.40]
Right Hip	5.23 [4.64, 5.96]	5.17 [4.84,5.51]	6.51 [6.16, 6.87]
Left Knee	6.26 [5.65, 7.01]	5.46 [5.09, 5.84]	5.05 [4.75, 5.35]
Right Knee	6.43 [5.84, 7.17]	5.60 [5.20, 6.01]	4.80 [4.50, 5.10]
Left Ankle	8.83 [8.18, 9.60]	4.68 [4.37, 5.00]	4.99 [4.66, 5.32]
Right Ankle	8.86 [8.23, 9.62]	5.06 [4.71, 5.41]	5.48 [5.15, 5.83]
Mid-Back	5.32 [4.72, 6.07]	5.38 [4.97, 5.80]	6.49 [6.18, 6.81]
Neck Base	5.30 [4.70, 6.05]	6.84 [6.36, 7.33]	6.26 [5.87, 6.68]
Left Shoulder	5.28 [4.66, 6.06]	6.73 [6.28, 7.19]	6.39 [6.05, 6.75]
Right Shoulder	5.21 [4.63, 5.94]	6.70 [6.23, 7.17]	6.26 [5.90, 6.63]
Overall	6.21 [5.61,6.95]	5.70 [5.33, 6.08]	5.93 [5.65, 6.21]

**TABLE 4.** Relative error between 3-dimensional trajectories of joints in single- and multi-camera output: mean RMSE as a percentage of maximum joint range of motion.

Keypoint	Treadmill Gait	Sit-to-Stand	Trunk Flexion-Extension
Left Hip	37.12	9.88	25.19
Right Hip	36.05	9.45	23.63
Left Knee	16.04	34.39	28.75
Right Knee	16.43	35.05	27.44
Left Ankle	12.22	157.72	199.65
Right Ankle	12.23	180.37	213.91
Mid-Back	38.66	8.73	23.59
Neck Base	37.09	9.42	11.44
Left Shoulder	33.83	9.79	13.23
Right Shoulder	33.06	9.73	12.84
Overall	27.27	46.45	57.97

**TABLE 5.** RMSE between key joint angles calculated from single- and multi-camera capture.

Joint Angle	Treadmill Gait [95% CI] (°)	Sit-to-Stand [95% Cl] O	Trunk Flexion-Extension [95% CI] (°)
Left Knee flexion	9.15 [8.86, 9.46]	9.68 [6.97, 12.95]	5.29 [5.02, 5.56]
Right Knee flexion	12.48 [12.20, 12.77]	10.62 [7.95, 13.84]	6.26 [5.95, 6.58]
Left Hip flexion	6.93 [6.76, 7.12]	14.63 [12.34, 17.38]	11.83 [11.36, 12.32]
Right Hip flexion	9.08 [8.91, 9.25]	15.40 [13.18, 18.08]	12.33 [11.90, 12.78]
Hip Rotation (axial plane)	2.30 [2.01, 2.70]	1.42 [1.32, 1.52]	1.88 [1.67, 2.14]
Shoulder Rotation (axial plane)	2.68 [2.38, 3.09]	2.53 [2.38, 2.68]	2.35 [2.16, 2.60]
Trunk Inclination	2.85 [2.71, 2.99]	8.75 [8.10, 9.42]	14.41 [13.84, 14.98]

**TABLE 6.** Intraclass correlation coefficients ICC(3,1) comparing ROM estimates from single- and multi-camera output.

Joint Angle	ROM ICC
Left Hip Flexion	0.990
Left Knee Flexion	0.938
Right Hip Flexion	0.985
Right Knee Flexion	0.933
Trunk Inclination	0.989
